# Rational Design of AAV-rh74, AAV3B, and AAV8 with Limited Liver Targeting

**DOI:** 10.3390/v15112168

**Published:** 2023-10-28

**Authors:** Christopher Chan, Kathryn K. Harris, Sergei Zolotukhin, Geoffrey D. Keeler

**Affiliations:** Division of Cellular and Molecular Therapy, Department of Pediatrics, University of Florida, College of Medicine, Gainesville, FL 32610, USA

**Keywords:** AAV, liver de-targeting, rational design

## Abstract

Recombinant adeno-associated viruses (rAAVs) have become one of the leading gene therapies for treating a variety of diseases. One factor contributing to rAAVs’ success is the fact that a wide variety of tissue types can be transduced by different serotypes. However, one commonality amongst most serotypes is the high propensity for liver transduction when rAAVs are administered peripherally. One of the few exceptions is the naturally occurring clade F AAV hematopoietic stem cell 16 (AAVHSC16). AAVHSC16 represents an interesting capsid in that it shows minimal liver transduction when injected peripherally. For capsids other than AAVHSC16, targeting non-liver tissues via peripheral AAV injection represents a challenge due to the high liver transduction. Thus, there is a demand for liver-de-targeted rAAV vectors. The rational design of rAAV capsids relies on current knowledge to design improved capsids and represents one means of developing capsids with reduced liver transduction. Here, we utilized data from the AAVHSC16 capsid to rationally design four non-clade F rAAV capsids that result in reduced liver transduction following peripheral injection.

## 1. Introduction

Gene therapy is recognized as a powerful tool for the treatment of a wide range of diseases [1]. Of all the gene therapy approaches, adeno-associated viruses (AAVs) are recognized as one of the safest, most effective methods for delivering therapeutic transgenes to a range of tissues. Recombinant AAV (rAAV) vectors are small, non-enveloped viruses that have had the rep and cap genes removed and replaced with therapeutic transgenes. Recombinant AAV represents an ideal gene delivery system as it does not cause a disease, cannot replicate, is minimally immunogenic, and minimally integrative while providing stable, long-term transgene expression [2,3]. This has led to rAAV vectors being utilized in 300 clinical trials that are either recruiting, soon to be recruiting, underway, or completed (ClinicalTrials.gov). Thus far, four AAV therapies have been approved by the Food and Drug Administration (FDA): Luxturna for the treatment of retinal dystrophy, Zolgensma for the treatment of spinal muscular atrophy, Hemgenix for the treatment of hemophilia B, and Elevidys for Duchenne muscular dystrophy (DMD) (fda.gov).

AAVs are a diverse group of viruses, with over 10 serotypes and 100 variants identified to date. Each serotype consists of unique capsid sequences that result in diverse tissue tropisms. The natural ability of AAV to be ‘targeted’ to specific tissues is responsible, at least in part, for AAV technology becoming the preferred gene delivery vehicle for many clinical applications. Unfortunately, though systemic AAV administration is preferred, it results in a relatively low therapeutic index [4]. This is due largely to off-target transduction occurring in the liver. To combat this, high doses of AAVs are used to achieve sufficient transduction in non-liver target tissue to treat disease. However, the use of high-dose AAVs has resulted in adverse side effects and has been implicated in playing a possible role in the death of six patients in clinical trials [5,6,7,8,9]. Thus, there is an urgent need to develop AAVs capable of efficiently transducing target tissues at low doses. An effective first step in developing these AAVs is de-targeting them away from the liver so that more viruses can transduce target tissues.

AAV serotypes are divided into ‘superfamilies’, or clades, based on phylogenetic relation. Clade F AAVs are comprised of AAV9 as well as the closely related hematopoietic-stem-cell-derived AAVs (AAVHSCs). Clade F members, except for one, utilize terminally exposed galactose for binding [10,11,12]. AAVHSC16 has been identified as showing reduced galactose binding compared to other clade F members [13]. Three residues at positions 501, 505, and 706 have been identified as playing a major role in the inability of AAVHSC16 to bind galactose and express transgenes [13]. Interestingly, AAVHSC16 results in reduced liver transduction in mice and non-human primates as compared to other clade F members and does not induce elevated liver enzyme levels in non-human primates at high doses, while maintaining robust tropism to other target tissues [13].

Here, we sought to determine if mutagenesis at residues corresponding to AAVHSC16 positions 501, 505, and 706 could be exploited in non-clade-F AAVs to decrease liver transduction. Four AAV serotypes were mutagenized at the respective residues and tested in this study: rh74, AAV3B, AAV6, and AAV8. Our results show that mutagenesis results in reduced transgene transduction and expression in liver tissue. These data support the idea that residues 501, 505, and 706 play an integral role in liver transduction in multiple AAV serotypes and may be used to de-target AAV therapies from the liver.

## 2. Materials and Methods

### 2.1. Animals and AAV Administration

Eight-week-old female C57BL/6J mice were purchased from The Jackson Laboratory. Mice were allowed to acclimate for one week in the vivarium prior to experimentation. Animals were then injected with 1 × 10^11^ (rh74 and rh74-16), 1 × 10^10^ (AAV8 and AAV8-16), or 1 × 10^9^ (AAV6 and AAV6-16) based on virus packaging efficiency. Animals remained in the vivarium for 2 weeks and were allowed food and water ad libitum. After 2 weeks, animals were euthanized and tissues harvested for downstream analyses.

### 2.2. Vector Production and Packaging Efficiency

rAAV-GFPs were produced and purified using a previously described standard protocol [14]. Briefly, HEK-293 cells were co-transfected with three plasmids: one derived from pACG-2 containing the AAV2 rep gene and one of the variant cap genes, one of the pscAAV-GFPs, and pHelper (Agilent, Santa Clara, CA, USA). AAVs were purified from both cells and culture medium 3 days later on double iodixanol gradient and quantified using qPCR.

### 2.3. In Vitro Studies

HuH7 cells were seeded in a 6-well dish at 75% confluence on the day of seeding. The next day, cells were infected with either rAAV3B-GFP or rAAV3B-HSC16-GFP at the M.O.I. of 10^3^. Two days post infection, cells were visualized for GFP fluorescence and then analyzed with FACS to quantify the transduction efficiency.

### 2.4. qPCR

A quantitative polymerase chain reaction (qPCR) was utilized to determine the transduction efficiency of AAV vectors following standard protocols. Briefly, animals were euthanized 2 weeks after AAV administration, tissue was harvested, and DNA was extracted with a 2-step protocol using (1) chloroform and (2) a Qiagen DNeasy kit (Hilden, Germany). SYBR GreenER (Thermo Scientific, Carlsbad, CA, USA) and transgene-specific primers (Eurofins genomics, Louisville, KY, USA) were used to determine the AAV vector genomes present in tissues. Linearized plasmid was used to make the standard curve used for quantification purposes. PCR was performed with a CFX96 Optics Module Thermal Cycler (Bio Rad, Hercules, CA, USA) for 40 cycles.

### 2.5. Immunofluorescence

Organs, including the liver, heart, gastrocnemius, brain, spinal cord, kidneys, and spleen, were harvested from rh74-, rh74-16-, AAV8-, AAV8-16-, AAV6-, and AAV6-16-treated mice. These organs were fixed in formalin and then embedded in paraffin. Tissues were sectioned using a Microtome at a thickness of 8 μm and placed onto glass slides. A Prolong Diamond Antifade Mountant with DAPI (Thermo Fisher, Eugene, OR, USA) was used to indicate tissue nuclei. The tissue was then imaged using a Keyence BZ-X800 Fluorescence Microscope (Osaka, Japan) to capture expression of the endogenous eGFP transgene and DAPI staining. GFP and DAPI expression was quantified with Fiji/ImageJ macro. Thresholds were set to identify pixels representing eGFP (transgene expression) and DAPI (nuclei). With this, the number of eGFP-positive cells were identified. Statistical analyses were completed using Prism 9. Images were prepared for publication in Photoshop.

### 2.6. Statistical Analyses

All data are represented as mean ± SEM. A Shapiro–Wilk test was used to determine data normality. For normally distributed data, an unpaired, two-tailed Student’s *t*-test was performed to determine significance. For non-normally distributed data, a Mann–Whitney test was used to identify significance. Groups were deemed to be significantly different at *p* ≤ 0.05. All statistical analyses were performed in Prism 9.

### 2.7. Study Approval

All animal studies were performed in accordance with the Florida Institutional Animal Care and Use Committee.

## 3. Results

### 3.1. Identification of Potential Candidates for Mutagenesis

**Figure 1:**
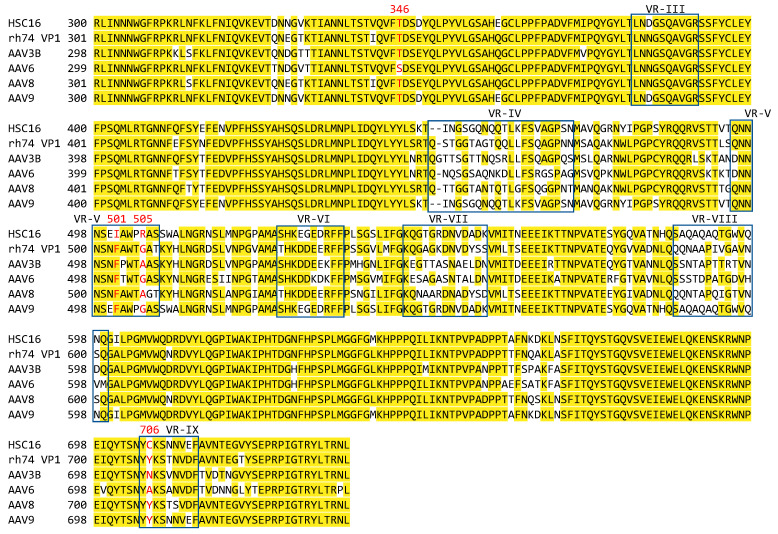
**AAV sequence alignments.** The capsid sequences of rh74, AAV3B, AAV6, and AAV8 were aligned with the sequences of AAVHSC16 and AAV9 to determine differences at positions 346, 501, 505, and 706.

AAVHSC16 differs from AAV9, and all other AAVHSCs, at residues 501 and 706, while also differing from the closely related AAVHSC15 at residue 346. In AAVHSC16, residue 501 is an isoleucine, while 501 in AAV9 and all other AAVHSCs is a phenylalanine. Interestingly, a gain-of-function I501F mutation did not fully restore binding in AAVHSC16 as compared to AAVHSC15, suggesting a possible role of other residues [13]. Residue 706 is a cysteine in AAVHSC16 while being a tyrosine in AAV9 and all other AAVHSCs. Residue 706 has been identified as the primary residue responsible for the reduced liver tropism associated with AAVHSC16 [13]. Residue 505 is used to define two groups of AAVHSCs: 505R (AAVHSC13, and 15–17) results in reduced galactose binding, while 505G (AAVHSC1, 3-4, and 6-9) enhances galactose binding. Thus, the respective residues corresponding to AAVHSC16 at positions 501, 505, and 706 may represent targets for mutagenesis to reduce liver tropism and expression in non-clade F AAVs. Sequence alignments were performed to identify non-clade-F serotypes that differed at residues 501, 505, and 706 from AAVHSC16. We identified four clinically relevant serotypes, rh74, AAV3B, AAV6, and AAV8, that shared the 501F with AAV9 but differed from AAVHSC16 at residues 505 and 706 (Figure 1). To determine the effects of these residues on liver tropism in non-clade-F AAVs, residues 501 (503 in rh74 and AAV8) were mutated to isoleucine, 505 (507 in rh74 and AAV8) were mutated to arginine, and 706 (708 in rh74 and AAV8) were mutagenized to cysteine in serotypes rh74, AAV6, and AAV8. In addition, a ‘singleton’ residue 345S in AAV6 was converted to Thr to make this position congruent with all other tested serotypes. The newly created serotypes were dubbed rh74-16, AAV3B-16, AAV6-16, and AAV8-16.

### 3.2. Packaging Efficiency

**Figure 2:**
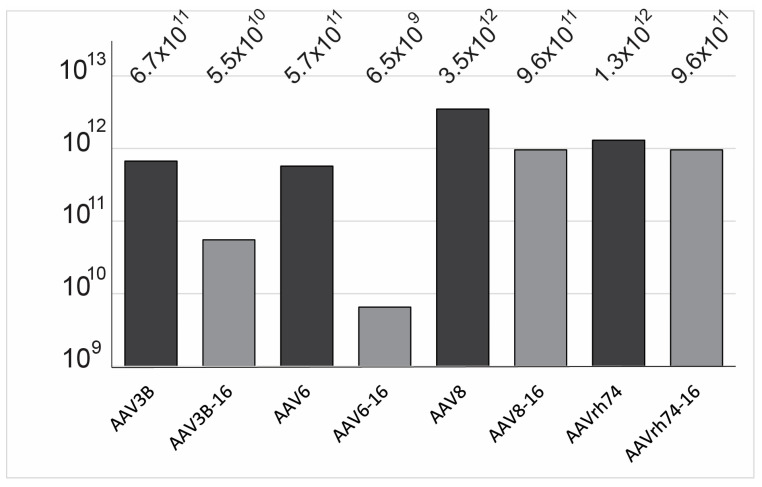
**Yields of iodixanol gradient-purified viral vectors incorporating scAAV-GFP expression cassettes.** The yield is provided as the total amount of the virus in genome-containing particles as determined by qPCR in purified and concentrated viral stock. The respective capsids incorporating the expression cassette are indicated below the bars. The wt capsids are shaded in black, and the HSC-16 mutants are shaded in grey.

Rationally designed variants adopting substitutions from other serotypes face potential handicaps related to their compatibility with the foster wild-type (wt) capsid structure. Their structural compatibility is usually assessed with the proxy parameter of the viral vector yield assessed side-by-side with the wt capsid. To test the structural fitness of the capsids, we prepared self-complementary (sc) rAAV-GFP vectors from 2 × 10^8^ cells for each of the parent serotypes and their respective de-targeting mutants. The yields of the wt AAV3B, AAV6, AAV8, and AAVrh74 serotype vectors were in general agreement with the field-accumulated knowledge where AAV8 yield was significantly higher compared to AAV3B or AAV6 (Figure 2). Across the board, all of the de-targeting HSC-16 mutants were less productive; for AAV3B-16 and AAV6-16, this reduction was more pronounced. These results corroborate the notion of the selective structural fitness pressure of the introduced AA residue motifs within a particular capsid scaffold that interferes with a higher yield vector production.

### 3.3. In Vitro Transduction Efficiency

Many AAV capsids have been identified as possible candidates for liver-directed AAV gene therapies due to high liver tropism in murine models. Unfortunately, many serotypes show reduced liver tropism in humans, making them less than ideal for clinical applications [15,16]. AAV3B has been identified as a potential candidate for improved liver-directed gene therapies in humans due to high liver tropism [17,18]. However, AAV3B shows minimal transduction efficiency in murine models [17]. For this reason, AAV3B and its mutant were assayed in vitro in the HuH7 human hepatocarcinoma-derived cell line. We have previously described using HuH7 for a directed evolution in vitro of AAV3B-derived combinatorial capsid libraries, showing that isolated variants efficiently transduced human hepatocytes in vivo, thus validating the model [19,20].

**Figure 3:**
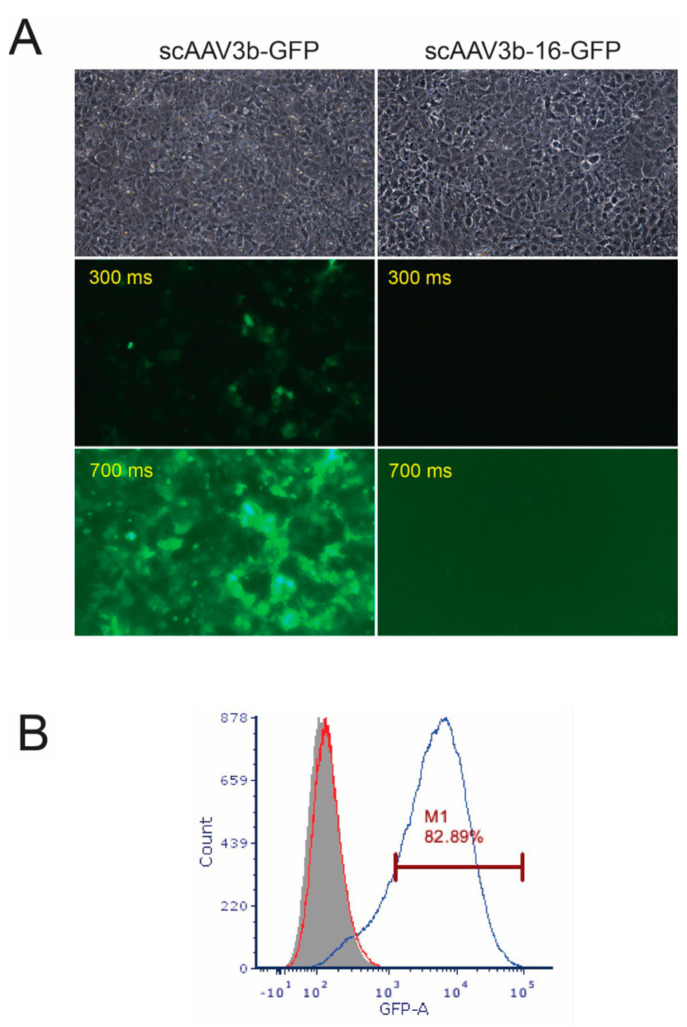
**Transduction assay in HuH7 cells in vitro for scAAV3B-GFP and its HSC-16 mutant.** Cells were infected at the MOI of 10^3^ and visualized one day post infection (**A**). Fluorescence intensities for the wt AAV3B (left column) and HSC-16 mutant (right column) were assayed with the same respective exposure times of 300 ms or 700 ms for comparison. The quantification of the transduction rate is shown in (**B**) as a FACS plot analysis where red represents non-transduced cells, grey represents AAV3B-16-transduced cells, and blue represents AAV3B-transduced cells.

HuH7 cells were infected with wt or mutant vectors at MOIs of 10^3^ and visualized on Day 2 after infection (Figure 3A). As expected, wt AAV3B-GFP transduced cells with notable efficiency, infecting over 80% of cells as quantified by FACS (Figure 3B). Even more impressive was the complete reversal of the transduction efficiency for the AAV3B-16 mutant, suggesting that the respective residues in AAV3B, corresponding to AAVHSC16 positions 501, 505, and 706, recognize the same galactose receptor and mediate liver targeting.

### 3.4. Capsid Mutagenesis Results in Reduced Liver Transduction and Expression in Non-Clade-F AAV Serotypes In Vivo

The AAV rhesus serotype 74 (rh74) is a Clade E member and is closely related to AAV8, another clade E member, sharing 93% homology [10,21]. The rh74 serotype was found in the rhesus macaque and was originally isolated from mesenteric lymph nodes and later from the spleen. The rh74 serotype is recognized for being highly efficient at transducing muscle in murine and non-human primate models when administered via the vasculature [22,23], while systemic administration results in less efficient transduction [24,25,26]. Furthermore, due to rh74 being isolated from non-human primates, it has been postulated that the incidence of pre-existing antibodies in patients will be reduced compared to other AAV serotypes, essentially increasing the pool of patients able to receive treatment [27,28]. This has been corroborated by studies showing rh74 to have one of the lowest seropositivity rates of all serotypes, with ~86% of all DMD patients tested being seronegative for anti-rh74 antibodies [24,29].

**Figure 4:**
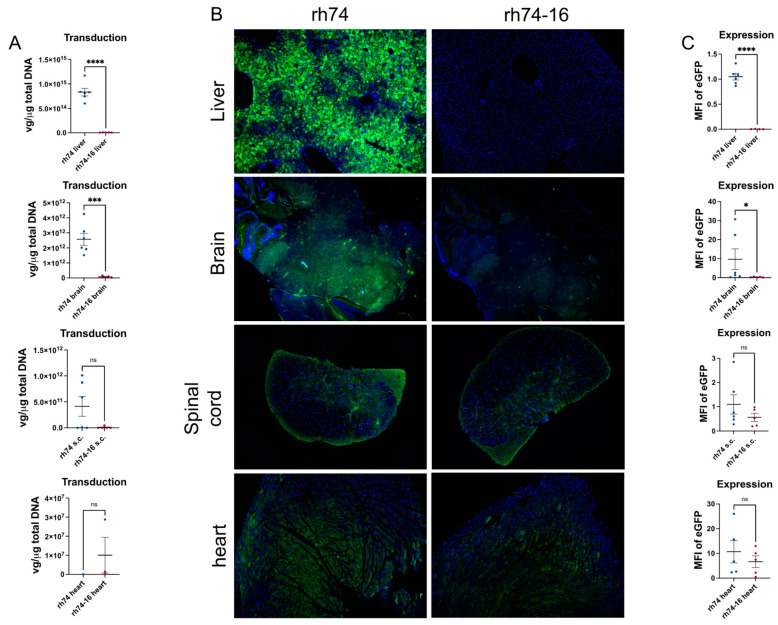
**rh74-16 transduction and expression efficiency.** The rh74-16 capsid resulted in reduced vector transduction efficiency in liver and brain (**A**). eGFP transgene expression was also significantly reduced in liver and brain in rh74-16-treated animals as compared to rh74-treated animals (**B**,**C**). No significant difference was noticed in transduction or expression levels in spinal cord or heart between the two capsids (**A**–**C**). Data represented as mean ± SEM. Transduction data are expressed as vector genomes per micrograms of total DNA. For immunofluorescent images, green = eGFP transgene and blue = nuclei. Expressions are represented as mean fluorescent intensity. N = 6 for rh74- and 5 for rh74-16-treated animals. **** *p* < 0.0001, *** *p* < 0.0004, * *p* < 0.05

To determine the effects of mutated residues 501, 505, and 706 on rh74 tropism, animals were peripherally injected with either rh74 or rh74-16, containing an eGFP transgene, via the tail vein. The rh74 and rh74-16 packaged efficiently enough to allow for 1 × 10^11^ vg to be administered to each mouse. Two weeks following vector administration, tissue was harvested for analyses of transduction and expression efficiencies. Quantitative polymerase chain reaction (qPCR) was utilized to determine the transduction efficiency of each vector. Results were normalized to the μg of total DNA analyzed. For transgene expression studies, fluorescent imaging was utilized to determine the amount of eGFP present in the tissue. Our results show that the rh74-16 vector results in significantly reduced liver transduction, ~114-fold lower (*p* < 0.0001) as compared to rh74 (Figure 4A). This suggests that mutations at residues 501, 505, and 706 may be effective at de-targeting rh74 from the liver. The rh74-16 vector also resulted in ~32-fold lower (*p* < 0.0004) transduction in the brain as compared to rh74 (Figure 4A). Likewise, rh74-16 resulted in significantly reduced transgene expression in the liver (*p* < 0.0001) and brain (*p* < 0.05) as compared to rh74 (Figure 4B,C). There was no significant difference in the transduction or expression efficiency of rh74 and rh74-16 in the spinal cord or heart (Figure 4A–C). Finally, transgene expression was equal between rh74 or rh74-16 in gastrocnemius, kidney, or spleen tissues (Figure S1), while no transduction was identified.

**Figure 5:**
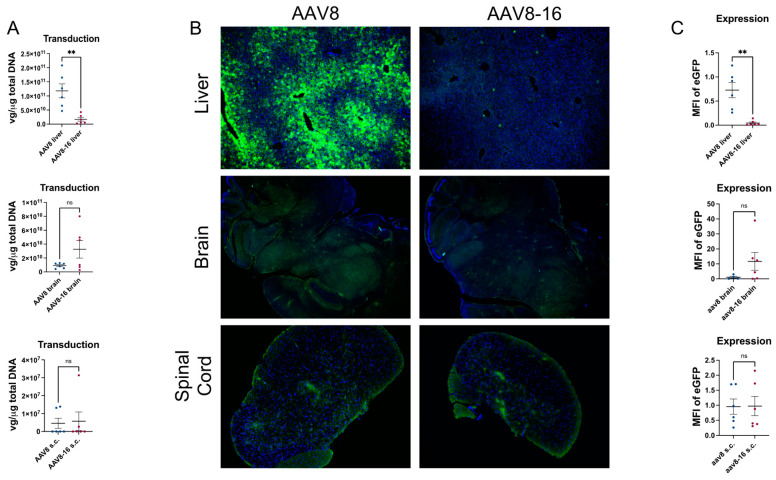
**AAV8-16 transduction and expression efficiency.** Use of the AAV8-16 capsid resulted in reduced vector transduction efficiency in (**A**). Transgene expression was significantly decreased in the liver of AAV8-16-treated animals as compared to AAV8-treated animals (**B**,**C**). No significant difference was noticed in transduction or expression levels in the brain or spinal cord between the two capsids (**A**–**C**). Data represented as mean ± SEM. Transduction data are expressed as vector genomes per micrograms of total DNA. For immunofluorescent images, green = eGFP transgene and blue = nuclei. Expressions are represented as mean fluorescent intensity. N = 6 for AAV8- and AAV8-16-treated animals. ** *p* < 0.005

AAV8 is a clade E member and is an isolate from rhesus monkey tissue. AAV8 has gained popularity, arguably due to the high tropism of this serotype for mouse liver tissue. In fact, in a mouse model, AAV8 was reported to have a ~50-fold greater liver cell transduction as compared to AAV2 [30]. This has resulted in AAV8 being the ‘gold standard’ for pre-clinical, liver-directed gene therapy studies in mice. Within clinical trials, AAV8 is utilized in ~40% of blood disorder trials using AAVs, thus representing the most commonly used serotype in this area [31]. Given the high tropism of AAV8 to liver tissue in mice, we sought to determine if HSC-16 capsid mutations would result in liver de-targeting. To determine this, mice were peripherally injected with 1 × 10^10^ vg/mouse via the tail vein with either AAV8 or AAV8-16 containing an eGFP transgene. In the liver, AAV8-16 resulted in a significantly reduced, ~7-fold lower (*p* < 0.005) transduction efficiency as compared to AAV8 (Figure 5). Expression levels of eGFP were also significantly lower in the livers of AAV8-16-treated animals as compared to AAV8-treated animals (*p* < 0.002) (Figure 5). These results suggest that the AAV8-16 vector was efficiently de-targeted from the liver. There was no differential transduction or expression identified between AAV8 and AAV8-16 in the brain or spinal cord (Figure 5). For heart, gastrocnemius, kidney, and spleen, transgene expression was equal between AAV8 and AAV8-16 tissue (Figure S2), but no transduction was identified.

**Figure 6:**
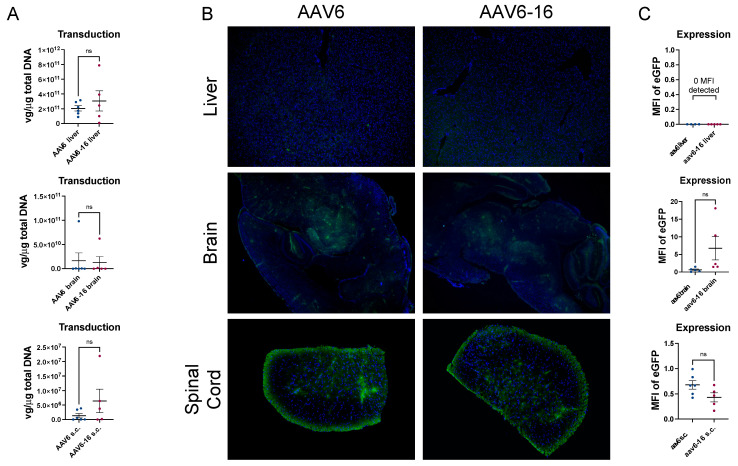
**AAV6-16 transduction and expression efficiency.** Both AAV6 and AAV6-16 capsids resulted in minimal transduction in liver, brain, and spinal cord (**A**). Likewise, transgene expression levels were low for liver, brain, and spinal cord (**B**,**C**). Data represented as mean ± SEM. Transduction data are expressed as vector genomes per micrograms of total DNA. For immunofluorescent images, green = eGFP transgene and blue = nuclei. Expressions are represented as mean fluorescent intensity. N = 6 for AAV6- and n = 5 for AAV6-16-treated animals.

AAV6 is a clade A member that was first isolated from a human adenovirus preparation [32]. AAV6 is closely related to AAV1 and AAV2, leading to the belief that it may have arisen from a homologous recombination between the two serotypes [32]. AAV6 has a broad tropism and has been identified as transducing cells in the heart, liver, skeletal muscle, and airway epithelium [17,33]. More recently, AAV6 was identified as being the most efficient AAV serotype for transducing human hematopoietic stem/progenitor cells (HSPCs) [34]. It has been argued that clade F members could mediate efficient HSPC transduction in the absence of DNA breaks [35]. This claim was refuted by Rogers et al. [36], who showed that AAV6 is far superior to clade F members at transducing HSPCs. This was corroborated by a separate, independent study by Dudek et al. that also showed AAV6 to be superior to clade F members for transducing HSPCs [37]. Here, we sought to determine if the mutated AAV6 vector would result in liver de-targeting as was seen with rh74-16 and AAV8-16. One more mutation (S346T) was introduced into the AAV6 wt backbone to make it consistent with AAV-HSC16 (Figure 1). Animals were i.v. injected with 1 × 10^9^ vg/mouse of AAV6 or AAV6-16 containing an eGFP transgene. For AAV6-16, due to the low virus yield, the utilized dose was a limiting factor for both the wt and HSC-16 mutant. Even with an overall low transduction rate, AAV6-16 showed no significant difference in transduction efficiency in the liver, brain, or spinal cord as compared to AAV6 (Figure 6). Likewise, minimal to no eGFP expression was identified. It is unclear if this is due to the mutagenesis being ineffective in AAV6 or if it is due to the low dose that was administered. Similar to the other two serotypes tested, no transduction from AAV6 or AAV6-16 was detected in the heart, gastrocnemius, kidney, or spleen, but equal expression was identified (Figure S3).

## 4. Discussion

Gene therapies aim to restore or modify cellular functions through the introduction of a therapeutic transgene. AAV technology has established itself as one of the premier gene therapy approaches and has become the leading platform for treating a variety of human diseases. AAVs are a diverse group of viruses, either naturally occurring or designed, with each serotype having specific tissue tropisms. The ability of different serotypes to target different tissues is one reason for the success of the AAV platform. The liver is the largest solid organ in the body and plays a role in detoxifying blood, digestion, metabolism, and immunity, and is also where blood clotting factors are produced. Further, the liver, in many respects, acts like a sponge and has been shown to be the main infection site for AAV [10]. Thus, the liver has long been one of the primary targets for AAV gene therapies. Hemophilia B represents the prototypical liver disease treated with AAV therapies. Hemophilia B results from decreased production of the clotting factor, factor IX (FIX), in the liver. FIX is small enough that protein replacement can be readily achieved via AAVs targeted to express FIX in liver cells.

While beneficial at times, the high propensity for AAVs to transduce liver cells is problematic when treating diseases where the target tissue is not the liver. The CNS, skeletal muscle, and heart represent three non-liver tissues that are currently being targeted by AAV therapies in clinical trials (Clinicaltrials.gov). DMD is one of the most widely recognized diseases for which an AAV therapy exists. DMD is an X-linked neuromuscular disease caused by mutations in the DMD gene and results in the absence of the cytoskeleton protein dystrophin. In this instance, high liver transduction levels are problematic, as this vastly reduces the amount of transgene making it to the target tissue. To date, high vector loads have been administered to overcome this hurdle. However, high vector loads are associated with adverse side effects and have recently been implicated in the death of multiple patients in clinical trials [5,6,7,8,9]. This suggests that the use of high AAV doses may not represent an effective strategy for overcoming undesired liver transduction. DMD represents just one example where the target tissue for therapeutic transgene delivery is not the liver. The issue of high AAV liver transduction is a hurdle that must be overcome to successfully treat all diseases where the liver is not the target tissue and peripheral administration will be utilized.

Much effort has been devoted to developing liver-de-targeted capsids. Modifying the AAV capsid has become the leading technique utilized to develop liver-de-targeted vectors. Rational design of AAV capsids relies on current scientific knowledge of AAVs to alter vector performance. In a recent report, Smith et al. found that AAVHSC16 minimally transduces liver tissue and that residues 501, 505, and 706 play a critical role in the propensity of clade F AAVs to transduce liver tissue [13]. In AAVHSC16, residue 501 is an isoleucine, is close to the galactose binding pocket, and has been identified as being a key residue responsible for the lack of galactose binding by AAVHSC16 in vitro [13]. An F501I mutation in AAVHSC15 resulted in reduced galactose binding, while a gain-of-function I501F mutation in AAVHSC16 partially restored galactose binding [13]. Though 501 mutations resulted in binding modulations, they were incomplete, suggesting other residues may play a role. Interestingly, a cysteine was found at residue 706 in AAVHSC16, though this is not the case for other clade F members, including AAVHSC15, which shows robust liver transduction. The authors discovered that making a Y706C mutation in AAVHSC15 resulted in reduced liver transduction [13]. Further, a C706Y mutation in AAVHSC16 largely rescued the ability of the serotype to transduce liver tissue [13]. Within AAVHSCs, residue 505 is occupied by either an arginine or a glycine. This residue has been shown to play a role in the efficiency of AAVHSCs in binding galactose, with those serotypes containing 505G being more efficient than serotypes containing 505R. This is thought to be due to the arginine being larger and thus creating steric hindrance that interferes with galactose binding [13,38]. Further, the arginine adds a positive charge, which also plays a role in inefficient galactose binding. Here we sought to determine if mutating residues corresponding to AAVHSC-16, namely 501, 505, and 706, could be utilized to de-target non-clade-F AAVs from the liver. We rationally designed new capsids for rh74, AAV3B, AAV6, and AAV8, mutagenizing the respective residues, and named them rh74-16, AAV3B-16, AAV6-16, and AAV8-16. Due to the poor liver transduction associated with AAV3B in mice, the AAV3B-16 capsid was evaluated in vitro. The de-targeting mutant AAV3B-16 was unable to transduce HuH7 hepatocarcinoma cells, a previously validated proxy human liver model cell line, while the wt AAV3B was very efficient, transducing more than 80% of these cells at the same MOI of 10^3^.

The remaining capsids were all tested in vivo. Our data show that introducing mutations into the AAV-rh74 and AAV8 serotypes at the positions corresponding to AAVHSC-16 (501I, 505R, and 706C) resulted in significantly reduced transduction in the liver. This highlights the importance of select AAV capsid residues in determining tropism.

Mutagenizing AAV6 in the same positions, as well as a S346T mutation, resulted in a significantly reduced structural fitness of the capsid and low yield of the virus. Whether this was due to this additional mutation residing inside the capsid scaffold remains to be investigated. Notwithstanding, the data indicate that not all AAV serotypes can be rationally modified to reduce their liver tropism, at least for this subset of the galactose-binding residues. As one would expect, alternative combinatorial approaches of directed evolution should be considered to achieve similar goals.

Reducing the liver transduction of AAV is a crucial first step in developing effective AAV therapies for targeting non-liver tissue. Here, we present three novel capsids that are effectively de-targeted away from the liver. The current study is limited in the fact that it is unclear what tissue these new viruses are targeting. In an effort to answer this question, we probed multiple tissues outside of the liver. Interestingly, we did not find a ‘new’ target tissue but rather found there to be no difference or in fact decreased transduction and expression in other tissues. However, we did effectively develop three new liver-de-targeted AAV capsids and thereby provided the groundwork for future studies. More specifically, these AAVx-16 capsids will be utilized in future work as backbones for directed evolution studies to develop new variants to target the tissues of choice. In the case of DMD, these capsids will provide an effective place to start in developing a capsid with decreased liver transduction and increased muscle transduction. The development of such a capsid may provide a means to deliver higher amounts of therapeutic compounds to muscle tissue at reduced AAV concentrations.

The research presented here supports previous data that highlighted the importance of residues 501, 505, and 706 for galactose binding and liver tropism in AAVHSCs, as well as providing evidence that these residues may be important for tropism regardless of the AAV clade [13,39,40,41]. De-targeting AAV away from the liver represents one of the largest hurdles facing peripherally administered AAVs where the liver is not the target. The AAVx-16 capsids presented here may represent a means of effectively de-targeting AAVs away from the liver and deliver therapeutic transgenes to other tissue. This provides the groundwork for developing AAVs that more specifically, and effectively, target non-liver tissue and may result in improved therapies with reduced adverse side effects in clinical trials.

## Data Availability

Data will be made available to the public upon any reasonable request.

## Supplementary Materials

The following supporting information can be downloaded at: https://www.mdpi.com/article/10.3390/v15112168/s1, Figure S1: rh74 and rh74-16 expression in gastrocnemius, spleen, kidney; Figure S2: AAV8 and AAV8-16 expression in gastrocnemius, spleen, kidney; Figure S3: AAV6 and AAV6-16 expression in gastrocnemius, spleen, kidney.
