# Supplementary material for: Rational Design of AAV-rh74, AAV3B, and AAV8 with Limited Liver Targeting
Source: Viruses. 2023 Oct 28;15(11):2168. doi: 10.3390/v15112168 (PMC10675213; doi:10.3390/v15112168)
Supplement: Supplementary file 1 [file viruses-15-02168-s001.zip › viruses-2647405-supplementary.pdf]

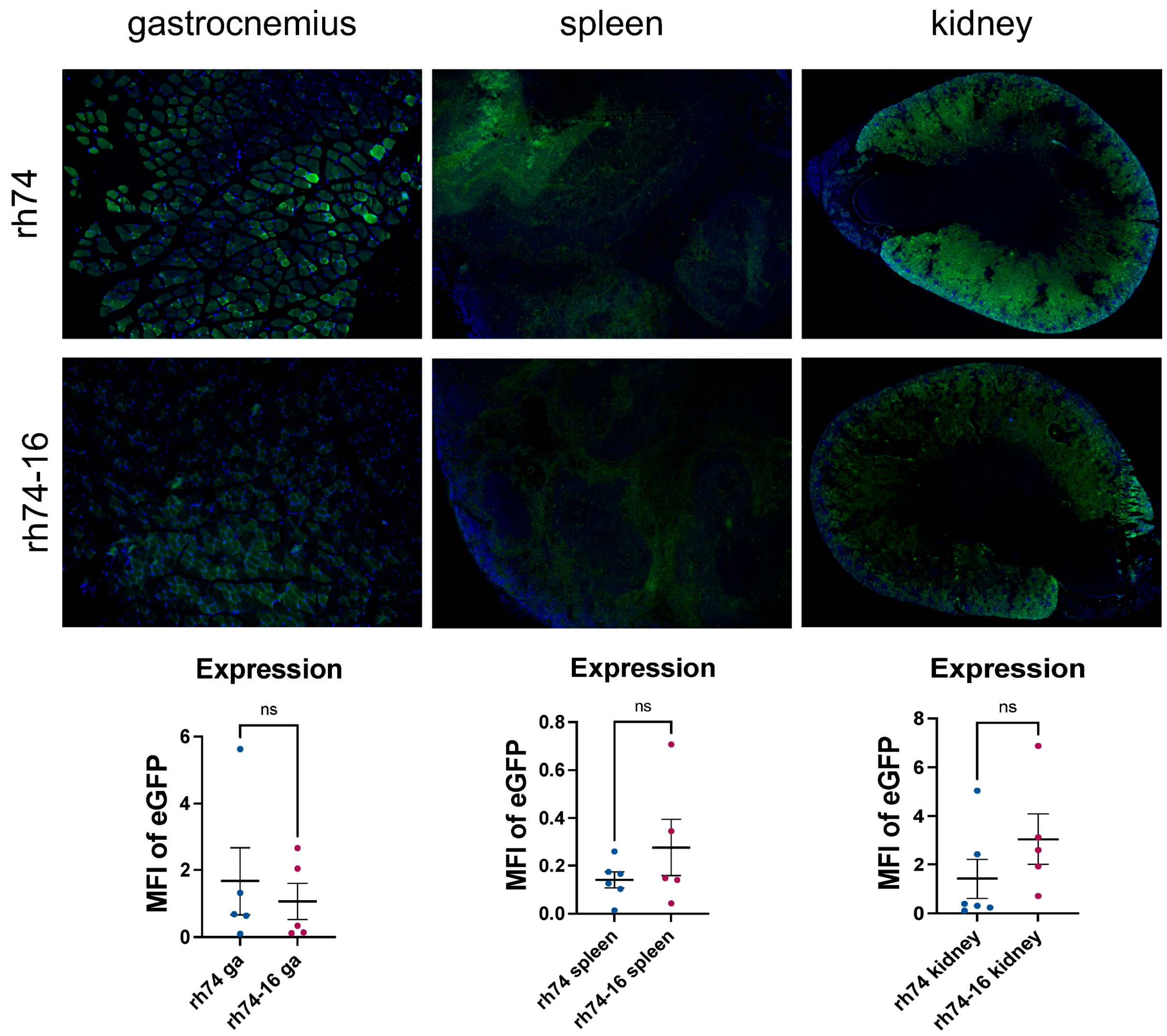

Figure S1. rh74 and rh74-16 expression in gastrocnemius, spleen, kidney. Transduction was not detected in the gastrocnemius, spleen, or kidney of animals. The rh74-16 and rh74 capsids also resulted in minimal transgene fluorescent. Data represented as mean ± SEM. For immunofluorescent images, green=eGFP transgene and blue=nuclei. Expression are expressed as mean fluorescent intensity. N=6 for rh74 and 5 for rh74-16 treated animals.

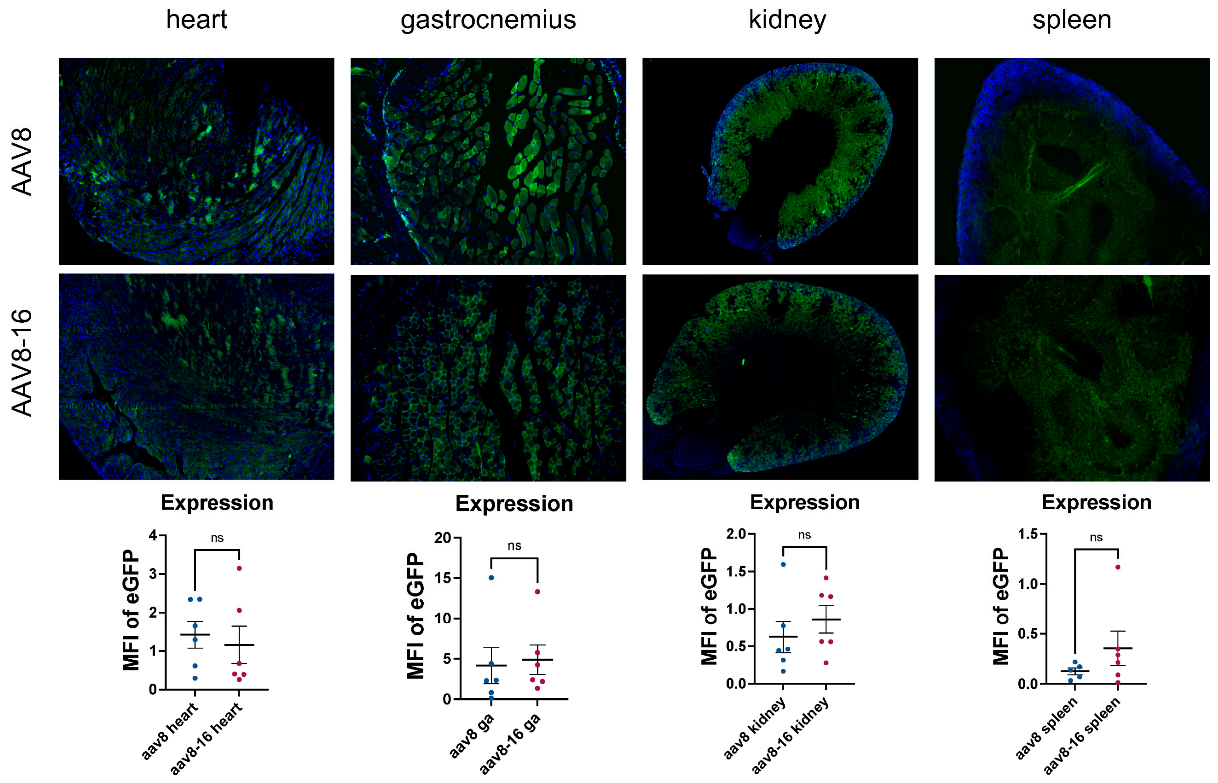

Figure S2. AAV8 and AAV8-16 expression in gastrocnemius, spleen, kidney. Transduction was not detected in the heart, gastrocnemius, kidney, or spleen, of AAV8 or AAV8-16 treated animals. Further, neither capsid resulted in significant transgene expression. For immunofluorescent images, green=eGFP transgene and blue=nuclei. Expression are expressed as mean fluorescent intensity. N=6 for AAV8 and AAV8-16 treated animals.

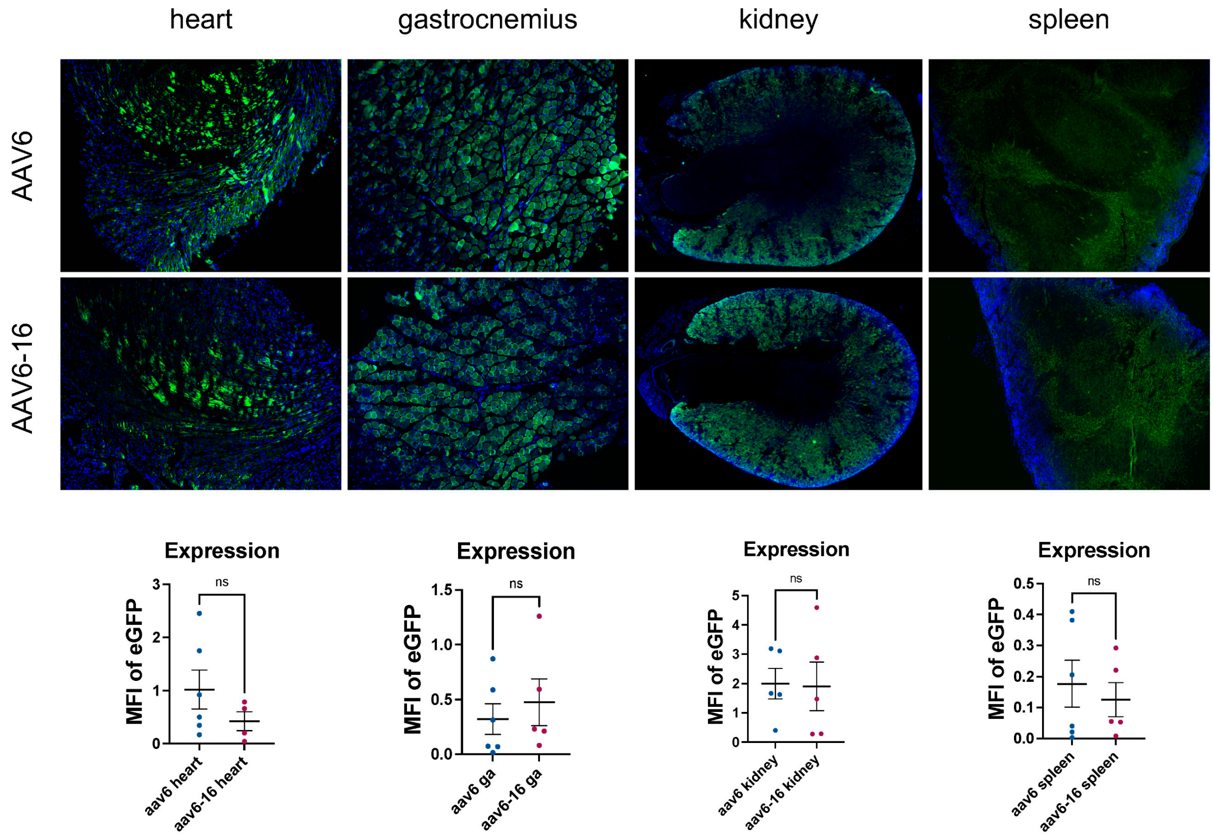

Figure S3. AAV6 and AAV6-16 expression in gastrocnemius, spleen, kidney. Transduction was not detected in the heart, gastrocnemius, kidney, or spleen for either AAV6 or AAV6-16 treated animals. This correlated with minimal eGFP fluorescence/transgene expression. For immunofluorescent images, green=eGFP transgene and blue=nuclei. Expression are expressed as mean fluorescent intensity. N=6 for AAV6 and n=5 for AAV6-16 treated animals.
